# Entropy of Financial Time Series Due to the Shock of War

**DOI:** 10.3390/e25050823

**Published:** 2023-05-21

**Authors:** Ewa A. Drzazga-Szczȩśniak, Piotr Szczepanik, Adam Z. Kaczmarek, Dominik Szczȩśniak

**Affiliations:** 1 Department of Physics, Faculty of Production Engineering and Materials Technology, Czȩstochowa University of Technology, 19 Armii Krajowej Ave., 42200 Czȩstochowa, Poland; ewa.drzazga@pcz.pl; 2 Institute of Pricing and Market Analysis, Analitico, 49/8 Królewska Str., 47400 Racibórz, Poland; pszczepanik@instytut-analitico.pl; 3 Department of Theoretical Physics, Faculty of Science and Technology, Jan Długosz University in Czȩstochowa, 13/15 Armii Krajowej Ave., 42200 Czȩstochowa, Poland; adam.kaczmarek@doktorant.ujd.edu.pl

**Keywords:** entropy, volatility, information theory, econophysics, sudden events, war, time series, data science

## Abstract

The concept of entropy is not uniquely relevant to the statistical mechanics but, among others, it can play pivotal role in the analysis of a time series, particularly the stock market data. In this area, sudden events are especially interesting as they describe abrupt data changes with potentially long-lasting effects. Here, we investigate the impact of such events on the entropy of financial time series. As a case study, we assume data of the Polish stock market, in the context of its main cumulative index, and discuss it for the finite time periods before and after outbreak of the 2022 Russian invasion of Ukraine. This analysis allows us to validate the entropy-based methodology in assessing changes in the market volatility, as driven by the extreme external factors. We show that some qualitative features of such market variations can be well captured in terms of the entropy. In particular, the discussed measure appears to highlight differences between data of the two considered timeframes in agreement with the character of their empirical distributions, which is not always the case in terms of the conventional standard deviation. Moreover, the entropy of cumulative index averages, qualitatively, the entropies of composing assets, suggesting capability for describing interdependencies between them. The entropy is also found to exhibit signatures of the upcoming extreme events. To this end, the role of recent war in shaping the current economic situation is briefly discussed.

## 1. Introduction

In general, sudden or extreme events translate to the atypical patterns and deviations from the expected observations. As such, the ability to detect and address accordingly aforesaid anomalies is of great importance in various areas of science, technology, or even social studies [1,2,3,4,5]. This is to say, timing and occurrence of sudden events is essential when considering reliability of a system under extreme external conditions. A special attention to these aspects is given in the field of economy, where sudden events correspond to a notable incline/decline in economic activity or may even mark a breakdown of some economic models, e.g., by exposing their limitations in terms of efficiency and rationality of the market [6]. In what follows, it is crucial to account for such events during economic modeling when considering processes such as the forecasting, decision making, or anomaly detection [7]. This is conventionally carried out on the grounds of the time series analysis, a vital part of data science [8]. The main reason for that is related to character of the time series itself, which are derived from the financial data and intrinsically encode information about economic events [9]. Thus, to allow discussion of the extreme changes in economy, appropriate tools in the time series domain are required.

In the context of the above, entropy appears as an intriguing analytical concept, which spans beyond its original field of thermodynamics. While in terms of the statistical mechanics, this property relates to the discrete probabilities of microstates, in the area of time series entropy, it is considered as an extension of the information theory [10,11,12,13], in accordance with the groundbreaking works by Shannon and Kolmogorov [14,15]. In particular, entropy can quantify the uncertainty, disorder, or simply randomness of the time series, without adding constraints on the corresponding probability distribution [13,16,17,18]. Hence, it constitutes an attractive alternative to the standard deviation for measuring market volatility [19,20]. However, entropy allows for discussing not only the magnitude of such fluctuations but also their distributions and patterns [21,22]. It can account for the nonlinearities and correlations in the datasets, simultaneously capturing interdependence between assets [23,24,25]. Moreover, since volatility relates to the degree of an asset movement over time, the corresponding entropy should be inherently sensitive to the sudden events or the economic shocks of interest. As a result, entropy constitutes potentially highly relevant framework for discussing impact of sudden events on the market and a pivotal tool in econophysics [10,12,13,18,26].

So far, the studies on the economic sudden events in terms of entropy have been limited mainly to a few instances, such as the investigations related to the 2008 economic crisis [27] or to the outbreak of the COVID-19 pandemic [11]. However, recent Russian invasion of Ukraine resulted in a yet another prominent economic shock, which is well defined in terms of the timeframes, and influences multiple market branches [28]. The economic consequences of this event constitute not only a perfect platform to investigate the impact of the shock of war on the modern economy but also to validate the entropic methodology in assessing market changes due to the extreme external factors. These arguments, along with the aforementioned general characteristics of entropy in the field of econophysics, constitute intriguing motivation to analyze this new measure in terms of the market volatility description and the resulting potential for the detection of sudden events. Herein, we provide our contribution to this still not fully explored area. In detail, we concentrate our study on the behavior of the main cumulative index of the Polish stock market (WIG20) and conduct our calculations with respect to the conventional Shannon entropy. The WIG20 index was chosen due to the direct proximity of the corresponding market to the theater of war as well as the relatively high development of the Polish economy. For convenience, the obtained results are compared with the predictions of the standard deviation. This analysis allows us to verify efficiency and predictive capabilities of the entropy-based formalism and to outline pertinent perspectives for the future research. It also provides the possibility to give preliminary insights into the other factors potentially influencing the WIG20 index, besides the pivotal shock of war.

## 2. Methodology

The present analysis is conducted for the time series of the daily log-returns, as calculated based on the financial data of interest. In particular, the daily log-returns ($R_{i}$) are derived by following the relation:

(1) $$R_{i}=\ln\frac{P_{i}}{P_{i-1}}\approx \frac{P_{i}-P_{i-1}}{P_{i-1}},$$

where $P_{i}$ ($P_{i-1}$) is the closing price of an asset on day *i* (i−1). In this manner, we obtain convenient time series data which are additive and symmetric in accordance with the scope of the present analysis. While the former property simply means that the log-returns are additive over time, the second one is much less self-explanatory. In brief, the symmetry of log-returns relates to the fact that positive and negative log-returns of equal magnitude are equidistant from zero on the logarithmic scale, yielding no net change when compared.

The volatility of the above time series is explored based on the two measures, namely, the standard deviation and the entropy. The former parameter is given by:

(2) $$\begin{array}{c} S=\sqrt{\frac{1}{N-1}\sum_{i=1}^{N}{\left(R_{i}-\mu \right)}^{2}}, \end{array}$$

for the *N* data points and μ being the arithmetic mean of all the returns. On the other hand, the latter measure is calculated based on the Shannon entropy [14]:

(3) $$\begin{array}{c} H=-\sum_{i=1}^{M}p_{i}\ln p_{i}. \end{array}$$

In Equation (3), *M* stands for the number of bins (known also as the *intervals* or *classes* [29]) in the discrete probability density function of the returns and $p_{i}$ is the probability related to a given bin. Herein, $p_{i}$ is calculated by employing the Riemann approximation as follows:

(4) $$\begin{array}{c} p_{i}=\left(x_{i+1}-x_{i}\right)f\left(x_{i+1}\right), \end{array}$$

where $x_{i}$($x_{i+1}$) is the left (right) width endpoint of a bin and $f(x_{i+1})$ denotes the corresponding height. Note that, in Equation (3), when the logarithm base is *e*, the entropy is measured in *nats*. One can also use base equal to 2 or 10, resulting in the units of *shannons* or *hartleys*, respectively. Obviously, the change of units does not influence the qualitative behavior of entropy.

In the present study, the above theoretical model is fed with the financial data of the WIG20 cumulative index and its composing stocks, as divided into two one-year-long datasets. The first set corresponds to the one-year timeframe before the invasion (24 February 2021–23 February 2022), whereas the second considers a similar period but after the beginning of the invasion (24 February 2022–23 February 2023). In the following, we arrive with the total of N=251 data points for each set, providing sufficient economic perspective for our calculations. Note that the WIG20 index serves here as a pivotal parameter for comparison between the two approaches in modeling volatility. However, due to its cumulative character, this index measures only the total fluctuations, and to gain better insight into the underlying correlations of the market, the composing stocks are discussed. All of these stocks, including the WIG20 index, are listed in Table A1 along with their full names, market symbols, and the basic summary statistics in Appendix A. This list is valid for the assumed-here time period but it is obviously subject to changes in the future. For the sake of completeness, it is also crucial to note that the component company Pepco was introduced to the stock market on 26 May 2021, i.e., the corresponding records do not cover the entire one-year period before the invasion. In addition, the composition of the WIG20 index changed four times over the analyzed timeframe of two years. In detail, on 18 March 2022, the already-mentioned Pepco and other company named mBank replaced previously indexed stocks of Tauron and Mercator, respectively. Similarly, on 16 September 2022, the company Kȩty replaced Lotos, and on 16 December 2022, Kurk switched with the PGING. All the described changes are appropriately marked in the Section 3 and in Appendix A.

To this end, for the purpose of the present study, both the datasets of interest are divided into the finite number of bins, which compose the discrete probability density function of the returns. There is no general and valid rule that determines the number and character of such bins [29]. The final choice is always strongly related to the population of data points and their variability. In general, one should never stay with the empty bins or decrease their number to the point that resolution of the probability distribution is too low. In reference to the multiple models for the bin number, we observe that M=20 is optimal for our case. In the first place, the chosen *M* value provides relatively high resolution of the probability distribution on equal footing across all considered time series, allowing us to not hinder information about the tails in some of the instances. Secondly, the assumed number of bins does not simultaneously exceed the upper theoretical limits for *N* ∼ 250, as set by the Velleman formula [29].

## 3. Results

**Figure 1:**
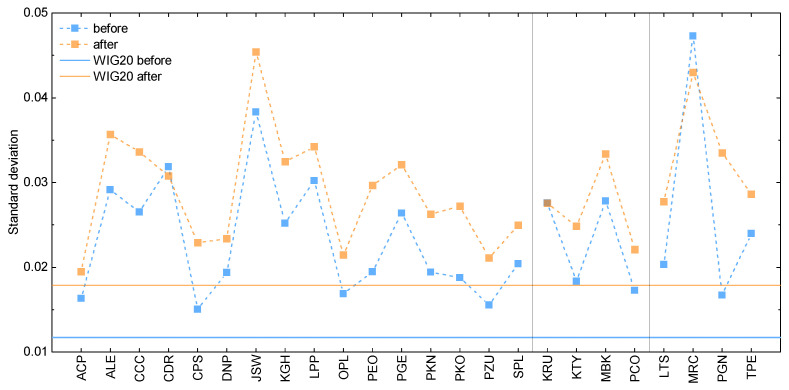
The standard deviation for the WIG20 index and its composing stocks. The first panel is for the constant component companies and the second (third) for the stocks introduced to (removed from) the index at some point. The results are given for the one-year time period before the beginning of the Russian invasion of Ukraine (blue) and after this event (orange). The solid lines correspond to the WIG20 index, whereas closed symbols represent estimates for the component stocks. Dashed lines are the guide for an eye.

In Figure 1, we depict standard deviation as calculated for the WIG20 index and its composing stocks. According to the initial assumptions, the results are presented here for the one-year timeframe before (orange) and after (blue) the beginning of the invasion. Note that Figure 1 is divided into three panels: the first for the constant component companies, whereas the second (third) panel corresponds to the stocks, which at some point were introduced to (removed from) the WIG20 index. For convenience, the corresponding numerical results and the percentage difference between estimates obtained for the two considered timeframes are given in Table A2 in Appendix A.

Upon the analysis of Figure 1, the total standard deviation appears to be higher after the beginning of the invasion than for the time period before it. This means that the volatility of the market visibly increases for the former dataset. In other words, this indicates higher degree of stock price variations in the second considered period, which can be caused by not only the decline but also incline of the asset value. Similar behavior can be observed for most of the composing stocks. In detail, one can notice that only companies such as Kruk (debt management and purchase), Mercator (medical devices), and CD Projekt (video game developer and publisher) do not comply with this trend. The first company shows practically indistinguishable values for the two considered datasets, while the two latter ones present inverse behavior in comparison to the total standard deviation. The observed standard deviation for the first two companies is potentially related to the fact that their stocks were not included in the WIG20 index for the entire time, meaning their impact on the total index was limited. Moreover, Mercator capitalization, as a producer of medical gloves, was heavily reduced by the end of the COVID-19 pandemic. Finally, the value of CD Projekt was subject to turbulence due to the mixed reviews of their flagship video game product Cyberpunk 2077. Thus, the standard deviation for each of the three companies is the results of not only the wartime market changes but also other, external factors. Despite these deviations, it can be stated that most of the composing assets as well as the cumulative results highlight the impact of the shock of war.

Nonetheless, the results for the individual stocks still allow us to observe that the largest volatility increase is present for the bank sector, with other notable examples in petroleum and telecommunication sectors (see Table A2 in Appendix A for details). Interestingly, by comparing the component estimates with the results for the cumulative index, we can note that the total standard deviation measure does not average values obtained for the individual stocks. In fact, this measure is always lower than any of the corresponding component values. This is true for both considered sets of data and can originate from the way that the cumulative index is calculated or potentially from the shortcomings of the standard deviation approach.

**Figure 2:**
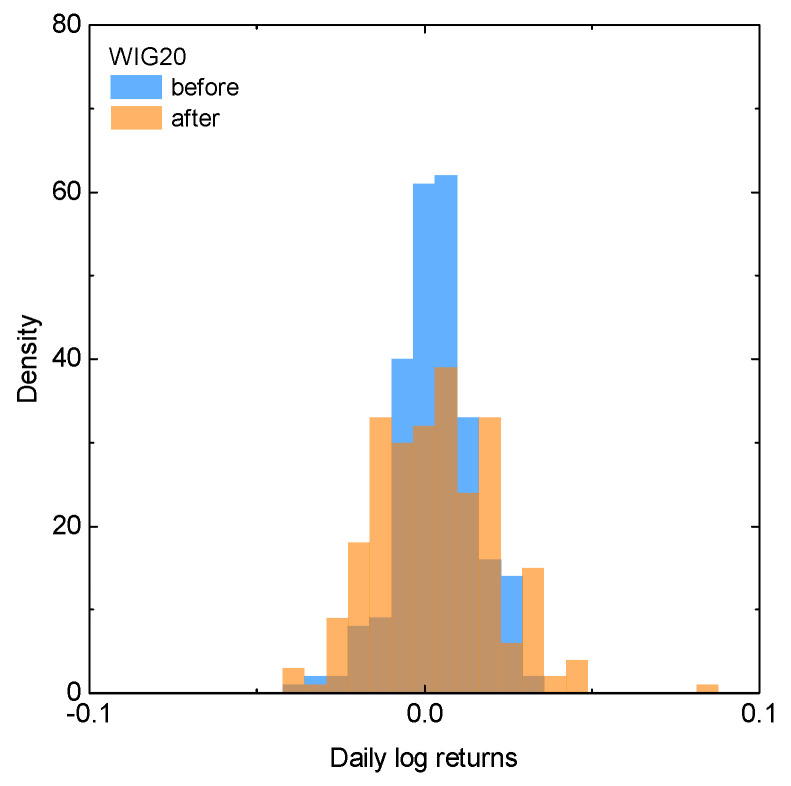
The discrete probability density function for the WIG20 index, for the one-year period before (blue) and after (orange) the beginning of the Russian invasion of Ukraine.

**Figure 3:**
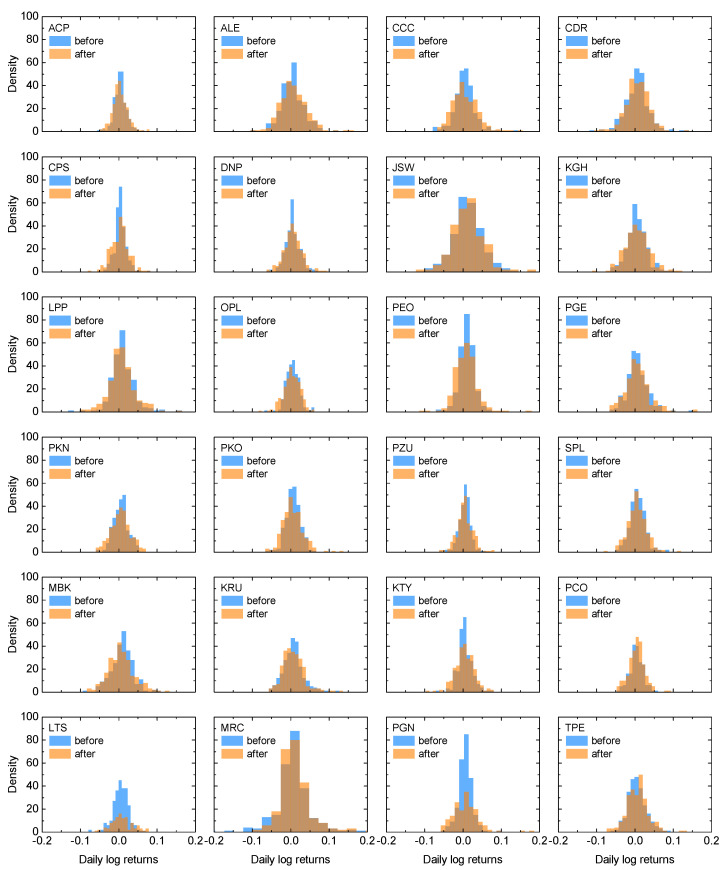
The discrete probability density function for the component stocks of the WIG20 index. The first four rows are for the constant component companies, and the fifth (sixth) row is for the stocks introduced to (removed from) the index at some point. The results are presented for the one-year time period before the beginning of the Russian invasion of Ukraine (blue) and after this event (orange).

To investigate more in detail the already observed trends, in Figure 2 and Figure 3 we present the discrete probability density function of the returns for the total index and its composing stocks, respectively. Note that these are the empirical distributions of the pooled returns. All the distributions are given for the time period before and after the beginning of the invasion, with the same color scheme as before. Based on Figure 2, it can be observed that the probability distributions for the WIG20 index resemble normal distribution. However, the wartime dataset is characterized by the fatter tails and lower central maxima than the distribution corresponding to the index values before the conflict outbreak. This observation is in qualitative agreement with the results obtained within the standard deviation approach, which suggest higher volatility of the market after the beginning of the invasion. The situation is once again similar when inspecting return distributions for the component stocks, i.e., volatility for most of the stocks is higher after the beginning of the Russian invasion. Still, there is some visible exception from this trend in terms of Pepco data. This is potentially due to the fact that, as mentioned earlier, data for Pepco do not cover the entire year before the invasion because of its relatively late introduction to the market on 26 May 2021.

**Figure 4:**
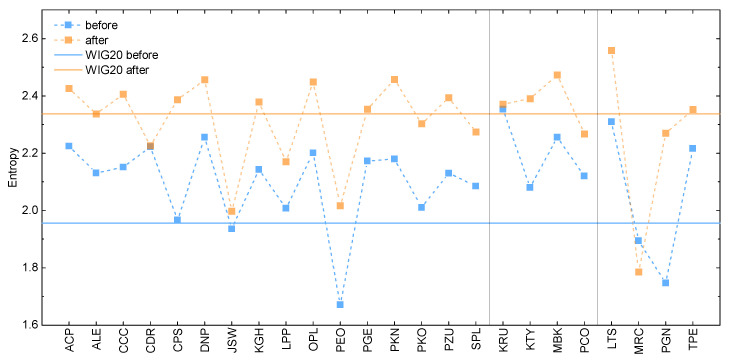
The Shannon entropy for the WIG20 index and its composing stocks. The first panel is for the constant component companies and the second (third) for the stocks introduced to (removed from) the index at some point. The results are given for the one-year time period before the beginning of the Russian invasion of Ukraine (blue) and after this event (orange). The solid lines correspond to the WIG20 index, whereas closed symbols represent estimates for the component stocks. Dashed lines are the guide for an eye.

It is next instructive to compare all the above results with the predictions of the entropic model. These are presented in Figure 4, in the form of the entropy estimates for the WIG20 index and its component stocks, based on the two types of the datasets of interest. In general, the total entropy, as well as the relative behavior, between composing entropies is similar to the standard deviation predictions. However, closer inspection of the results allows us to observe that, contrary to the previous case, here, all the component stocks exhibit higher entropy after the war outbreak. The only exception is Mercator, relatively late in the WIG20 index and experiencing the COVID-19-related problems during the entire analyzed period, as described before. Moreover, this time, the results for the total index qualitatively average results for the component companies. The mentioned observation is particularly visible for the data corresponding to the timeframe after the beginning of the invasion. The results allow us also to note that the percentage difference between the results before and after invasion is smaller for each of the calculated entropies than in the case of the standard deviation results (see Table A2 in Appendix A for details). Finally, the obtained entropies appear to follow the character of the discrete probability density function in Figure 2 and Figure 3, in terms of the differences between results obtained for the two considered timeframes.

**Figure 5:**
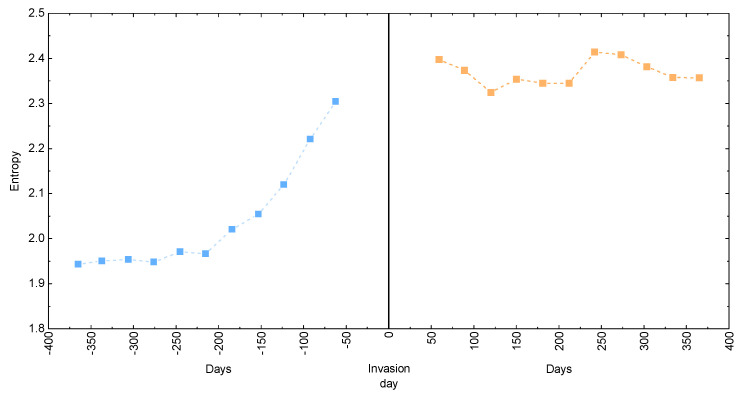
The Shannon entropy for the WIG20 index as calculated for different periods of time before (blue) and after (orange) the beginning of the Russian invasion of Ukraine. Dashed lines are the guide for an eye.

To supplement our analysis, we additionally plot the entropic index of WIG20 index for various time periods within the here-assumed timeframes. In Figure 5, we present the obtained results for the datasets before (left panel) and after (right panel) the beginning of the considered conflict. Both panels depict different behavior, namely, before the invasion, the entropic index clearly increases when the assumed time distance from the invasion date becomes smaller. On the other hand, the entropy is relatively stable throughout the entire period after the invasion data, independent of the number of considered days.

## 4. Conclusions

In the present study, we validated the entropy-based theoretical framework in describing behavior of financial time series under the influence of sudden and extreme external events. This was carried out in the context of the WIG20 main cumulative index of the Polish stock market for a one-year-long data samples before and after the Russian invasion of Ukraine, respectively. In particular, it was shown that entropy reproduces some of the features of the standard deviation when describing the effects of the shock of war. The obtained results confirmed that entropy can indeed be used as an alternative measure of volatility. These findings not only agree with the previous studies on applications of entropy in finances [10,11,18,24,27], but also supplement them by considering the wartime-driven changes in the stock market. For convenience, all the numerical results are summarized in Table A2 in Appendix A.

In addition to the above, the present study reveled several noteworthy differences between entropy and standard deviation measures. First, the entropy was found to capture the character of empirical data in qualitative agreement with the discrete probability distribution function, which was not always the case when considering the standard deviation measure. As a result, it is concluded that the entropy was better in highlighting differences between results obtained for the two timeframes of interest. This was particularly visible in the case of CD Projekt data, where standard deviation predicted inverse behavior to the probability distribution function and entropy. Finally, it was also revealed that the entropy of cumulative index qualitatively averages entropies of the composing stocks, again in contrast to the standard deviation estimates. This finding is particularly interesting since it shows that entropy holds potential in encompassing interdependencies between assets.

The last part of the analysis revealed that the entropy measure can be used to quantify anomalies in time series toward their better detection. In particular, entropy exhibits different functional character when considering it for various time periods, before and after the beginning of the invasion. In other words, it can be argued that entropy shows signatures of the upcoming economic shock. That means that the impact of a potential sudden event can be visible in the entropy behavior when the time range is sufficiently small and the context data are available for a long time range. In the future, entropy may constitute a building block for future tools aimed at sudden (extreme) event prediction. Interestingly, these results also clearly indicate that the shock of war has a long-lasting effect of increased volatility of the market, at least within the one-year time perspective. To further verify the presented observations, we note that the analysis can be extended toward other more complex or larger datasets and be conducted via more sophisticated entropic models based not only on the Shannon entropy but also on other formulations, e.g., by Rényi [30] or Tsallis [31].

To this end, all the obtained results allow us to make some preliminary statements on the role of invasion in shaping the current economic situation in Poland. The calculated standard deviation and entropy measures clearly point out that the volatility of the Polish market is higher after the crisis outbreak than before. That is to say, the presented study allows us to conclude that the shock of war visibly impacts the Polish economy, according to the fact that the entire analysis was conducted with respect to this well-defined point in time. However, it is difficult to judge how big this impact is in comparison to other factors, such as the still-persisting effects of the COVID-19 pandemic or the internal economic decisions of the Polish government and related financial institutions e.g., in terms of changes in the interest rates of the National Bank of Poland [32]. To address the impact of an additional factors, besides the considered shock of war, extended investigations spanning beyond the scope of the present analysis are required. This can be carried out by identifying the aspects of influence and then by analyzing them separately, but on the same footing, within the economic model of choice. However, due to the potential complexity of the problem, it is argued here that the proposed analysis should incorporate a more sophisticated approach based, for example, on the network and behavioral modeling, in agreement with the recent insights from the field of complex systems [33]. Since similar observations can be made at the level of the European or even global market, it is expected that our entropic approach may provide interesting results with the dependence on the proximity to the conflict zone or the bond strength with the Ukrainian or Russian market. Still, such analysis will be limited in terms of influencing factors, and the above complex approach is expected to be also valuable for such large-scale simulations. In summary, the shock of war appears to be an important factor of recent economic turmoil, but its magnitude in the context of other factors is yet to be determined.

## Figures and Tables

**Figure A1:**
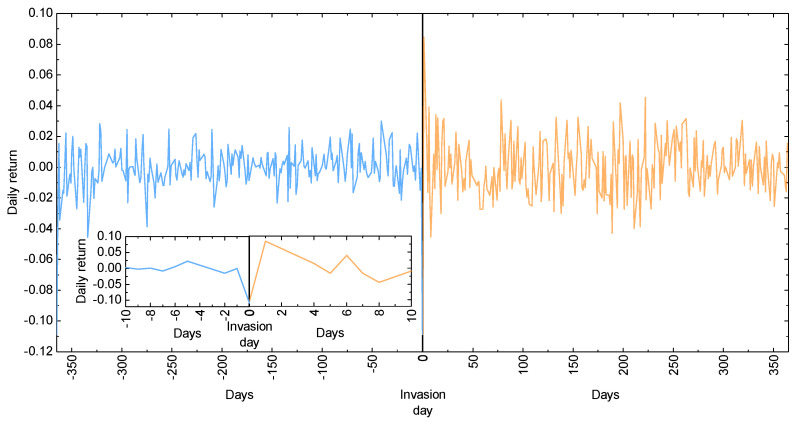
The daily log-returns for the WIG20 cumulative index before (blue) and after (orange) the beginning of the 2022 Russian invasion of Ukraine. For convenience, the inset presents data in the vicinity of the initial invasion day.

## Data Availability

Not applicable.

## Appendix A. Summary Statistics

The current Appendix section contains supplementary data to the discussion presented in the main text.

In Figure A1, the daily log-returns for the WIG20 cumulative index are depicted. The left panel presents data before the conflict outbreak (blue), whereas the right panel depicts data after the beginning of the invasion (orange). The inset presents a more detailed view in the vicinity of the initial invasion day. Qualitatively similar behavior can be observed for each of the WIG20 composing assets.

In Table A1, the basic summary statistics of the daily log-returns are given for the WIG20 cumulative index and its composing assets. The data are provided for two considered timeframes, i.e., one year before the invasion day and one year after this date. In Table A2, the numerical values of the calculated standard deviation and entropy, as obtained within the presented analysis, are collected. The data are given in a similar fashion as in Table A1.

**Table A1:** The summary statistics of the daily log-returns before (outside the brackets) and after (inside the brackets) the outbreak of the 2022 Russian invasion of Ukraine. The cumulative index data are followed by the first group for the constant component companies and then by the second (third) group for the stocks introduced to (removed from) the index at some point. The dates of the introduction/removal of the composing companies are given next to the name of the corresponding company.

Name	Symbol	Mean	Minimum	Maximum	Skewness	Kurtosis
Cumulative Index	WIG20	0.00027 (0.00017)	−0.0455 (−0.0452)	0.0299 (0.0844)	−0.2908 (0.5006)	1.1820 (1.3099)
Asseco	ACP	0.00074 (0.00056)	−0.0595 (−0.0488)	0.0608 (0.0765)	−0.0201 (0.4742)	1.7600 (1.3305)
Allegro	ALE	−0.0026 (0.0010)	−0.1123 (−0.1027)	0.1067 (0.1578)	0.1747 (0.5547)	1.1735 (1.9310)
CCC	CCC	−0.00196 (−0.00050)	−0.0851 (−0.068)	0.1327 (0.1490)	0.4102 (0.7795)	3.4547 (1.5699)
CD Projekt	CDR	−0.00118 (−0.00022)	−0.1257 (−0.1024)	0.1307 (0.1347)	0.1356 (0.0001)	2.3658 (1.6826)
Cyfrowy Polsat	CPS	0.00029 (−0.00150)	−0.0395 (−0.0847)	0.0729 (0.0782)	0.5066 (0.0800)	2.2896 (0.8452)
Dino	DNP	0.00076 (0.00157)	−0.0670 (−0.0673)	0.0659 (0.0909)	0.1096 (0.2339)	0.9815 (1.3584)
JSW	JSW	0.00144 (0.00251)	−0.1117 (−0.1345)	0.1170 (0.3130)	−0.0557 (1.5369)	0.5071 (9.5325)
KGHM	KGH	−0.00088 (0.00011)	−0.0673 (−0.1178)	0.0853 (0.1168)	0.0967 (0.2907)	0.6175 (1.2485)
LPP	LPP	0.00241 (0.00052)	−0.1393 (−0.1090)	0.1468 (0.1581)	0.3934 (0.3732)	4.5029 (2.1642)
Orange	OPL	0.00128 (−0.00021)	−0.0653 (−0.0870)	0.0578 (0.0572)	0.2096 (−0.3807)	1.3278 (0.6936)
Bank Pekao	PEO	0.00256 (−0.00016)	−0.0711 (−0.1221)	0.0625 (0.1714)	−0.3068 (0.7317)	1.5768 (5.6249)
PGE	PGE	0.00076 (0.00062)	−0.0721 (−0.0691)	0.1358 (0.1567)	0.6716 (0.9674)	2.6880 (3.5208)
Orlen	PKN	0.00070 (0.00042)	−0.0640 (−0.0620)	0.0457 (0.1096)	−0.0736 (0.2118)	0.2344 (0.6775)
Bank PKO	PKO	0.00167 (−0.00029)	−0.0718 (−0.0717)	0.0017 (0.1327)	−0.2323 (0.8032)	1.0216 (2.7165)
PZU	PZU	0.00053 (0.00084)	−0.0644 (−0.0660)	0.0523 (0.0784)	−0.3524 (0.2138)	1.7112 (1.4921)
Santander Bank	SPL	0.00190 (0.00035)	−0.0536 (−0.0867)	0.0823 (0.1143)	0.4418 (0.3425)	1.3168 (1.7196)
Pepco (18 March 2022)	PCO	−0.00050 (0.00074)	−0.0470 (−0.0523)	0.0558 (0.1143)	0.1075 (0.6496)	0.4942 (3.1461)
mBank (18 March 2022)	MBK	0.00275 (0)	−0.1002 (−0.0851)	0.0943 (0.1264)	−0.0907 (0.4069)	0.9970 (0.7190)
Kȩty (16 September 2022)	KTY	0.00084 (−0.00001)	−0.0684 (−0.1017)	0.0744 (0.0757)	0.3034 (−0.1884)	1.8525 (1.2969)
Kruk (16 December 2022)	KRU	0.00219 (0.00136)	−0.0622 (−0.0584)	0.1313 (0.1220)	0.9346 (0.9295)	2.7356 (2.2608)
Tauron (18 March 2022)	TPE	−0.00014 (0.00053)	−0.0617 (−0.0796)	0.0853 (0.1301)	0.4780 (0.5036)	0.6674 (2.1356)
Mercator (18 March 2022)	MRC	−0.00614 (0.00050)	−0.2348 (−0.0980)	0.2080 (0.2571)	0.0931 (1.9378)	5.0186 (7.6932)
LOTOS (16 September 2022)	LTS	0.00113 (0.00481)	−0.0826 (−0.0611)	0.0614 (0.0740)	−0.5117 (0.3371)	1.8753 (0.0411)
PGNiG (16 December 2022)	PGN	−0.00016 (0.00044)	−0.0603 (−0.0644)	0.0568 (0.1793)	−0.1985 (1.4098)	1.3151 (5.8434)

**Table A2:** The numerical values of the standard deviation and entropy, as calculated for the WIG20 index and its composing stocks. Similarly to Table A1, the cumulative index data are followed by the first group for the constant component companies and then by the second (third) group for the stocks introduced to (removed from) the index at some point. The dates of the introduction/removal of the composing companies are given next to the name of the corresponding company. The results are presented for the one-year time period before the beginning of the Russian invasion of Ukraine and after this event. For convenience, the percentage difference between estimates obtained for the two timeframes of interest is given for the standard deviation and entropy.

Name	Symbol	Standard Deviation Before	Standard Deviation After	Percentage Difference	Entropy Before	Entropy After	Percentage Difference
Cumulative Index	WIG20	0.012	0.018	40%	1.956	2.336	17.71%
Asseco	ACP	0.016	0.019	17.63%	2.224	2.426	8.68%
Allegro	ALE	0.029	0.036	21.54%	2.131	2.336	9.18%
CCC	CCC	0.027	0.034	22.95%	2.151	2.405	11.15%
CD Projekt	CDR	0.032	0.031	3.180%	2.223	2.226	0.14%
Cyfrowy Polsat	CPS	0.015	0.023	42.11%	2.387	1.967	19.29%
Dino	DNP	0.019	0.023	19.05%	2.256	2.456	8.49%
JSW	JSW	0.038	0.045	16.87%	1.936	1.998	3.15%
KGHM	KGH	0.025	0.032	24.56%	2.143	2.378	10.39%
LPP	LPP	0.030	0.034	12.50%	2.008	2.170	7.76%
Orange	OPL	0.017	0.021	21.05%	2.201	2.449	10.66%
Bank Pekao	PEO	0.019	0.030	44.90%	1.671	2.016	18.71%
PGE	PGE	0.026	0.032	20.69%	2.173	2.353	7.95%
Orlen	PKN	0.019	0.026	31.11%	2.180	2.460	12.07%
Bank PKO	PKO	0.019	0.027	34.78%	2.010	2.302	13.54%
PZU	PZU	0.016	0.021	27.03%	2.130	2.393	11.63%
Santander Bank	SPL	0.020	0.025	22.22%	2.085	2.274	8.67%
Pepco (18 March 2022)	PCO	0.017	0.022	25.64%	2.121	2.267	6.66%
mBank (18 March 2022)	MBK	0.028	0.033	16.39%	2.262	2.473	8.912%
Kȩty (16 September 2022)	KTY	0.018	0.025	32.56%	2.080	2.390	13.87%
Kruk (16 December 2022)	KRU	0.026	0.028	3.640%	2.353	2.371	0.76%
Tauron (18 March 2022)	TPE	0.024	0.029	18.87%	2.216	2.352	5.95%
Mercator (18 March 2022)	MRC	0.047	0.043	8.89%	1.894	1.785	5.93%
LOTOS (16 September 2022)	LTS	0.020	0.028	33.33%	2.310	2.558	10.19%
PGNiG (16 December 2022)	PGN	0.017	0.033	64%	1.747	2.269	26%
